# Augmented oxidative stress and reduced mitochondrial function in ageing goat testis

**DOI:** 10.1002/vms3.296

**Published:** 2020-07-06

**Authors:** Mohammed Majrashi, Ayaka Fujihashi, Mohammed Almaghrabi, Maali Fadan, Eddie Fahoury, Sindhu Ramesh, Manoj Govindarajulu, Haley Beamon, Chastity N. Bradford, Olga Bolden‐Tiller, Muralikrishnan Dhanasekaran

**Affiliations:** ^1^ Department of Drug Discovery and Development Harrison School of Pharmacy Auburn University Auburn AL USA; ^2^ Department of Pharmacology Faculty of Medicine University of Jeddah Jeddah KSA; ^3^ Department of Agricultural and Environmental Sciences Tuskegee University Tuskegee AL USA; ^4^ Department of Biology Tuskegee University Tuskegee AL USA

**Keywords:** ageing, goat, mitochondrial function, oxidative stress, testes

## Abstract

Recently, there is a significant increase in the commercial use of goat products. Nevertheless, there are very few reports on the characterization of redox biomarkers and mitochondrial function in the goat testis. Therefore, in this study we studied the markers of oxidative stress and mitochondrial functions in the goat testis during the process of ageing. Alterations in the markers of oxidative stress/redox biomarkers (contents of reactive oxygen species, nitrite, lipid peroxide, protein carbonyl, glutathione and activities of glutathione peroxidase, monoamine oxidase) and mitochondrial function (Complex‐I and Complex‐IV activities) were elucidated during the process of ageing. Augmented oxidative stress and decreased mitochondrial function were prominent during ageing in the goat testis. Ageing can lead to induction of oxidative stress and decreased production of ATP; however, the prooxidants generated must be effectively removed from the body by the innate antioxidant defence system to minimize the damage to the host tissue. Conversely, the antioxidants cannot completely scavenge the excessive amount of reactive oxygen species produced during ageing or pathological conditions leading to significant cell death and tissue damage. Thus, the use of effective and potent antioxidants in the feed could significantly reduce oxidative stress and improve mitochondrial function, resulting in enriched goat health.

## Graphical abstract

This study evaluates the cellular changes that occur in goat during ageing. Markers of oxidative stress and mitochondrial function validate the ageing process.

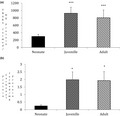

## 1 INTRODUCTION

Ageing is a natural process associated with time‐dependent gradual deterioration of the physiological functions necessary for survival and reproduction due to several endogenous and exogenous (environmental) factors. The precise underlying molecular mechanisms of ageing has not been elucidated; however, the increased production of free radicals and lifespan have a strong correlation indicating that the increased accumulation of reactive oxygen species (ROS) to be one of the primary mechanisms of ageing (Sergiev, Dontsova, & Berezkin, 2015). Ageing also affects the reproductive system and the associated decline in reproductive capacity could result from a combination of morphological and molecular alterations in the reproductive organs (Handelsman & Staraj, 1985). Gonads, especially testis, are of particular interest in ageing research due to decline of reproductive capacity. Furthermore, ageing of testis has direct implications for longevity, as decreased testicular function is associated with decreased longevity in several species (Partridge, Gems, & Withers, 2005). Literature on testicular function and its deficits during ageing has been, thus far, limited. However, several studies have demonstrated that aged testes undergo profound structural and morphological alterations (Frungieri, Calandra, Bartke, & Matzkin, 2018; Jiang et al., 2014).

The commercial business and recreational farming associated with domestic goat (*Capra aegagrus hircus*, family Bovidae) is developing substantially at large numbers and the various quality goat‐derived products are constantly being consumed around the world (Gall, 1996; Wilkinson & Stark, 1987). Due to the upswing in the comprehensive importance of goats, the goats must be raised healthily and have to be observed carefully to retain the well‐being of an individual goat and its herd (Jaudas, Mobini, & Jaudas, 2006). In addition to the declining reproductive function, ageing testes is associated with loss of muscle mass and concomitant increase in fat mass (Katznelson et al., 1996); decreased muscle strength (Martin, Farrar, Wagner, & Spirduso, 2000) and decreased bone mineral density (Riggs et al., 1982), all of which can adversely affect the well‐being. However, the relationship between oxidative stress and testicular ageing has not been investigated. Hence, this study was designed to demonstrate the influence of ageing on the testis of kiko goats in different age groups and to assess the role of oxidative stress and mitochondrial functions in the testis during ageing.

## 2 MATERIALS AND METHODS

### 2.1 Chemicals and reagents

Thermo Scientific Pierce 660 nm Protein Assay reagent kit was purchased from Thermo Scientific (Pierce) for protein quantification. Griess reagent was purchased from Thermofisher Scientific. 2,4‐Dinitrophenylhydrazine (DNPH) and nicotinamide adenine dinucleotide phosphate (NADPH) were purchased from Tokyo Chemical Industry America. Phosphate‐buffered saline (PBS), nicotinamide adenine dinucleotide (NADH), 2`, 7‐dichlorofluorescindiacetate (DCF‐DA), pyrogallol, phosphoric acid, O‐phthalaldehyde (OPT), L‐glutathione reduced, cytochrome c, bovine serum albumin (BSA) trichloroacetic acid and thiobarbituric acid were purchased from Sigma Aldrich.

### 2.2 Goat testis tissue

Kiko goats of three different age groups were obtained from commercial vendors. The sample size considered for each group was 5 (*n* = 5) in number applicable to all the three different age groups. Hence a total of 15 goats were used. A two‐sided *t*‐test was used and significance for statistical comparisons was set at *p* < .05. The three age groups include: Neonate (13–20 days), juvenile (108–124 days) and adult (over 6–9 months). The goats had free access to water. Medium quality forage (>10% protein) were provided. Does were fed a pelleted supplement (16% CP, 3.04 Mcal/kg of DE, as fed) at 454 g/d from kidding to weaning. The goats were slaughtered to collect their testes. The two testes of each of the 15 goats were carefully removed, labelled for proper identification and placed in liquid nitrogen after collection until further analysis. A prober amount of the testes corpus (body) was weighed, minced separately with addition of Halt™ Protease Inhibitor Cocktail. This was followed by homogenization with a high‐magnitude ultrasonic sonicator for 2 min with 2 ml PBS. The homogenate was further centrifuged at 12,000 RPM for one hour and the supernatant was collected to be used in experiments.

### 2.3 Reactive oxygen species (ROS) generation

The generation of reactive oxygen species generated in the testis of neonate, juvenile and adult groups was estimated via spectrofluorometry by measuring the conversion of non‐fluorescent chloromethyl‐DCF‐DA (2′, 7‐ dichlorofluorescindiacetate, DCF‐DA) to fluorescent DCF using an excitation wavelength of 492 nm and an emission wavelength of 527 nm. Results were expressed as percentage change from the control (Dhanasekaran, Tharakan, & Manyam, 2008; Katz et al., 2017; Zheng et al., 2014).

### 2.4 Nitrite content

Nitrite content in the testis of neonate, juvenile and adult groups was measured using Griess reagent. An azo product formed was measured spectrophotometrically at 545 nm (Giustarini, Dalle‐Donne, Colombo, Milzani, & Rossi, 2008).

### 2.5 Mitochondrial Complex‐I activity

NADH oxidation to NAD⁺ is catalysed by mitochondrial Complex‐I (NADH dehydrogenase). Tissue homogenate obtained from the testis of neonate, juvenile and adult groups was added to PBS and conversion of NADH to NAD⁺ was measured spectrophotometrically at 340 nm (Bhattacharya et al., 2018; Ramesh et al., 2018; Ramsay, Dadgar, Trevor, & Singer, 1986).

### 2.6 Mitochondrial Complex‐IV activity

Cytochrome C oxidation is catalysed by mitochondrial Complex‐IV (Cytochrome C oxidase). Cytochrome C oxidation was spectrophotometrically measured in the testis of neonate, juvenile and adult groups at 550 nm (Bhattacharya et al., 2018; Ramesh et al., 2018; Ramsay et al., 1986; Wharton & Tzagoloff, 1967).

### 2.7 Glutathione content

GSH content in the testis of neonate, juvenile and adult groups were measured via spectrofluorometry using O‐phthalaldehyde (OPT) condensation (Cohn & Lyle, 1966; Muralikrishnan & Mohanakumar, 1998; Zheng et al., 2014).

### 2.8 Glutathione peroxidase activity

Spectrophotometric method was used to measure glutathione peroxidase activity in the testis of neonate, juvenile and adult groups using NADPH as a substrate (Ahuja et al., 2017; Majrashi et al., 2018).

### 2.9 Lipid peroxidation

Lipid peroxide content formed in the testis of neonate, juvenile and adult groups was measured via colorimetry by measuring the malondialdehyde (MDA) content in the form of Thiobarbituric acid‐reactive substances (TBARS) (Bhattacharya et al., 2018; Dhanasekaran et al., 2007; Ohkawa, Ohishi, & Yagi, 1979; Zheng et al., 2014).

### 2.10 Protein carbonyl content

Protein carbonyl content in the testis of neonate, juvenile and adult groups were measured by the tagging of 2,4‐dinitrophenylhydrazine (DNPH) to the protein carbonyl groups which results in the formation of stable dinitrophenyl (DNP) hydrazones which can be quantified spectrophotometrically at 375 nm (Dalle‐Donne, Rossi, Giustarini, Milzani, & Colombo, 2003; Levine, Williams, Stadtman, & Shacter, 1994).

### 2.11 Monoamine oxidase (MAO) activity

Total monoamine oxidase activity in the testis of neonate, juvenile and adult groups was measured fluorimetrically by determining the amount of 4‐hydroxyquinoline formed as a result of kynuramine oxidation (Bhattacharya et al., 2018; Majrashi et al., 2018; Morinan & Garratt, 1985; Muralikrishnan & Mohanakumar, 1998).

### 2.12 Protein quantification

Protein was quantified using Thermo Scientific Pierce 660 nm Protein Assay reagent kit (Pierce, Rockford, IL). Bovine serum albumin (BSA) was used as a standard for protein measurement.

### 2.13 Statistical analysis

Data were reported as mean ± *SEM*. Statistical analyses were accomplished using one‐way analysis of variance (ANOVA) followed by Dunnet's multiple comparisons test (*p* < .05) and was determined to be statistically significant. The statistical analyses were performed using Prism‐V software (La Jolla, CA, USA). All the determinants were made in triplicates until otherwise mentioned.

## 3 RESULTS

General data: The average body weight of neonates was 3.06 ± 0.43 kg, juvenile was 19.5 ± 3.12 kg and adult age group was 43 ± 5.78 kg. No change in food consumption habits or any abnormal behaviour was noted. No statistically significant weight loss or any serious illness was noted in all the goats from different age groups.

### 3.1 Ageing increases reactive oxygen species and nitrite in goat testis

**FIGURE 1:**
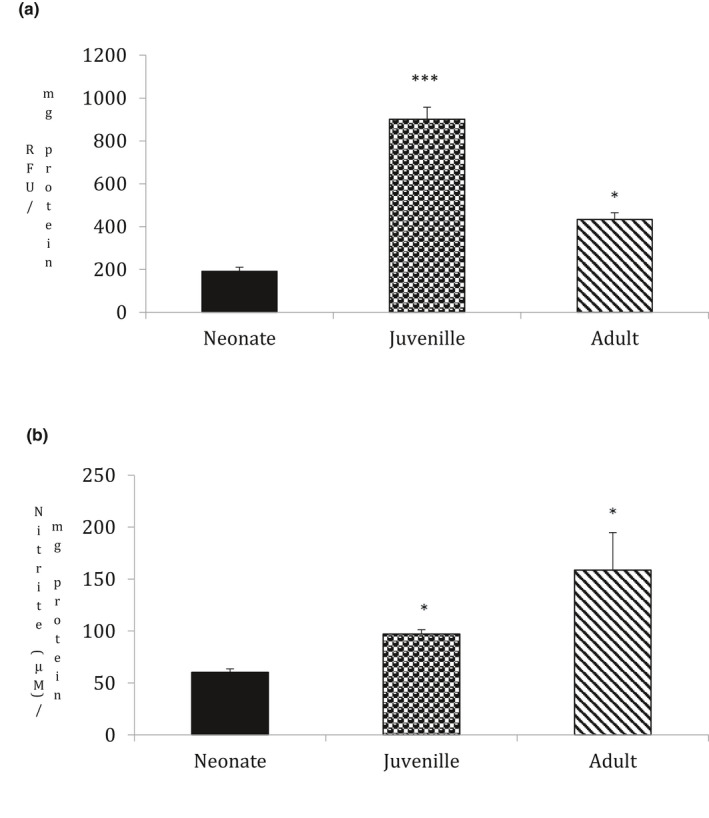
Statistical comparisons were made using one‐way ANOVA/Dunnett's multiple comparison test. Note (*) indicates a statistically significant difference when compared with controls. (a) Effect of ageing on ROS generation: ROS was measured spectrofluorimetrically. Ageing showed a significant increase in ROS generation (*p < .05, *n* = 5). ROS was measured as relative fluorescence units (492/527 nm)/mg. Results are expressed as (%) change as compared to the control ± *SEM*. (b) Effect ageing on Nitrite content: Nitrite content was measured spectrophotometrically. Ageing showed a significant increase in Nitrite content (*p < .05, *n* = 5). Nitrite content was measured as the absorbance of chromophoric azo product (545 nm) resulting from the reaction of NO_2_ with sulfanilamide under acidic condition. Results are expressed as (%) change as compared to the control ± *SEM*

The generation of reactive oxygen species (ROS) triggers oxidative stress and induces irreversible oxidation of lipids and proteins, which has lethal effects on cells leading to cell death. Statistically significant increase in ROS generation was noted in both juvenile (*p* = .003) and adult (*p* = .02) goat testis when compared with neonate group (Figure 1a, *n* = 5, *p* < .05). Similarly, the nitrite content significantly increased in both juvenile (*p* = .05) and adult group (*p* = .02) on comparison to the neonates (Figure 1b, *n* = 5, *p* < .05). This suggests that ageing significantly induces oxidative stress by increasing ROS and nitrite content.

### 3.2 Ageing inhibits mitochondrial function in goat testis

**FIGURE 2:**
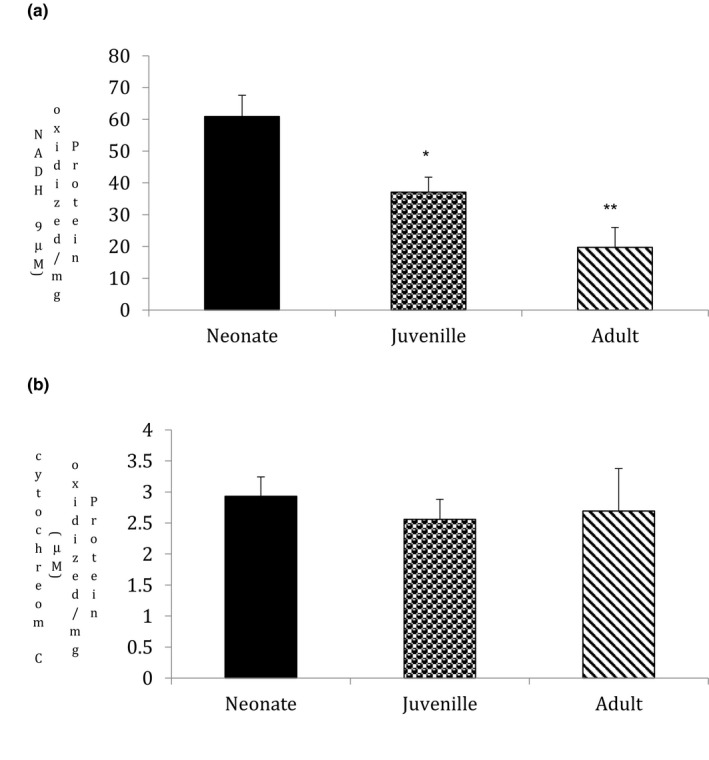
Statistical comparisons were made using one‐way ANOVA/Dunnett's multiple comparison test. Note (*) indicates a statistically significant difference when compared with controls. (a) Effect of ageing on Complex‐I activity: Complex‐I activity was measured spectrophotometrically using NADH as substrate. Ageing showed a significant decrease in Complex‐I activity (*p < .05, *n* = 5). Results are expressed as (%) change as compared to the control ± *SEM*. (b) Effect of ageing on Complex‐IV activity: Complex‐IV activity was measured colorimetrically using cytochrome‐C as substrate. Ageing did not affect Complex‐IV activity. Results are expressed as (%) change as compared to the control ± *SEM*

To explore the effects of ageing on mitochondrial function and to understand the molecular processes involved, we evaluated Complex‐I and Complex‐IV activity in three different groups. Ageing notably decreased mitochondrial function, as demonstrated by statistically significant decrease in Complex‐I activity in the juvenile (*p* = .035) and adults (*p* = .01) when compared with the neonate group (*n* = 5, *p* < .05; Figure 2a). However, our results showed that ageing did not affect Complex‐IV activity (*n* = 5, Figure 2b).

### 3.3 Ageing alters the activities of antioxidant enzymes in goat testis

**FIGURE 3:**
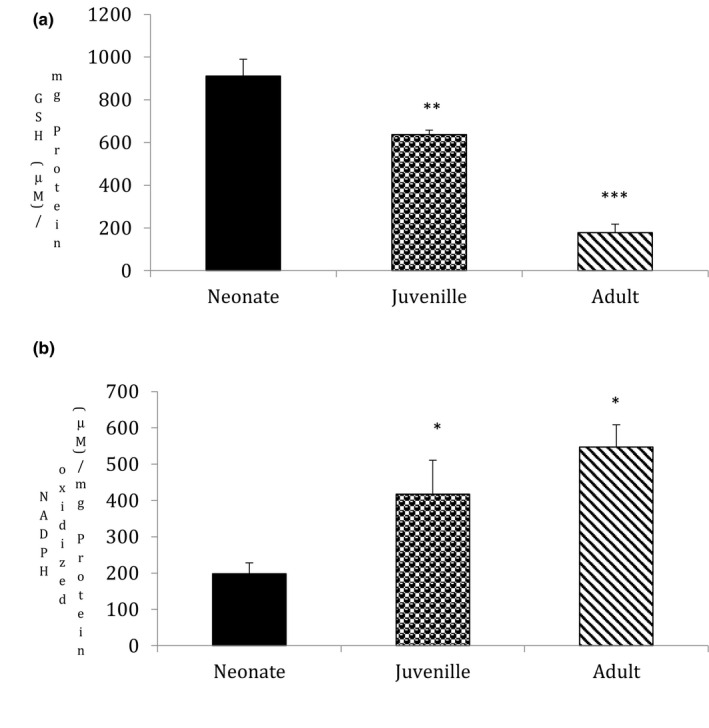
Statistical comparisons were made using one‐way ANOVA/Dunnett's multiple comparison test. Note (*) indicates a statistically significant difference when compared with controls. (a) Effect of ageing on glutathione content: Glutathione content was measured spectrophotometrically. Ageing showed a significant depletion in GSH content (*p < .05, *n* = 5). Results are expressed as (%) change as compared to the control ± *SEM*. (b) Effect of ageing on glutathione peroxidase activity: Glutathione peroxidase activity was measured spectrophotometrically using NADPH as substrate. Ageing showed a significant increase in glutathione peroxidase activity (*p < .05, *n* = 5). Results are expressed as (%) change as compared to the control ± *SEM*

A defence mechanism of the cell is to promote antioxidant expression and activity, which protects against highly reactive oxy or nitro radicals and their harmful toxic effects. We therefore investigated the effect of ageing on the activities of glutathione content and glutathione peroxidase activity. These play a significant role in scavenging toxic free radicals. Ageing significantly decreased glutathione content in juvenile (*p* = .04) and adults (*p* = .01) in comparison to neonates (Figure 3a, *n* = 5, *p* < .05) and interestingly increased glutathione peroxidase activity in both juvenile (*p* = .05) and adults (*p* = .05) on comparison to neonates (Figure 3b, *n* = 5, *p* < .05).

### 3.4 Ageing increases lipid peroxidation and protein carbonyl in goat testis

**FIGURE 4:**
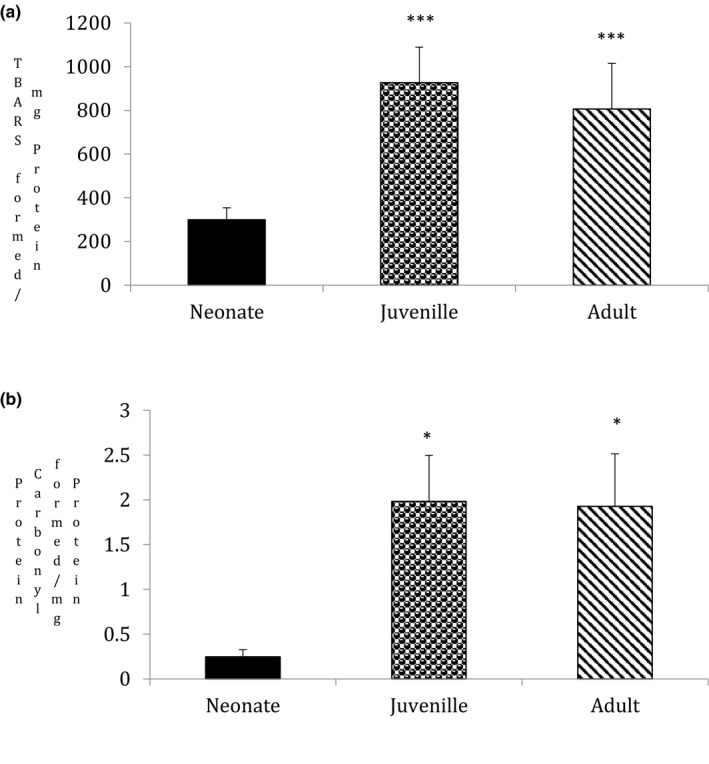
Statistical comparisons were made using one‐way ANOVA/Dunnett's multiple comparison test. Note (*) indicates a statistically significant difference when compared with controls. (a) Effect of ageing on lipid peroxide formation: Lipid peroxide was measured spectrophotometrically. Due to the increased ROS generation and Nitrite content, ageing induced a significant formation of lipid peroxide (*p < .05, *n* = 5). Lipid peroxide formation was measured as TBARS formed (532 nm)/mg protein. Results are expressed as (%) change as compared to the control ± *SEM*. (b) Effect of ageing on protein carbonyl content: Protein carbonyl content was measured spectrophotometrically at 375 nm. Ageing induced a significant increase in protein carbonyl content (*p < .05, *n* = 5). Protein carbonyl content was measured as DNP hydrazones formed/mg protein. Results are expressed as (%) change as compared to the control ± *SEM*

The generation of free radicals triggers oxidative stress and induces irreversible oxidation of lipids and proteins. Therefore, lipid peroxidation and protein carbonyl content were investigated in this study. Ageing significantly induced lipid peroxidation in juvenile (*p* = .009) and adults (*p* = .004) in comparison to neonates (Figure 4a, *n* = 5, *p* < .05). Similarly, protein carbonyl content increased in both juvenile (*p* = .02) and adult group (*p* = .03) on comparison to neonates (Figure 4b, *n* = 5, *p* < .05).

### 3.5 Ageing alters monoamine oxidase activity in goat testis

**FIGURE 5:**
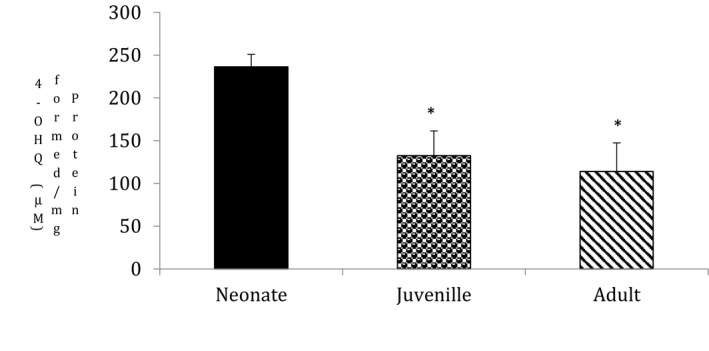
Effect of ageing on monoamine oxidase activity: Monoamine oxidase activity was measured spectrofluorimetrically using kynuramine as substrate. Ageing significantly decreased monoamine oxidase activity (*p < .05, *n* = 5). Results are expressed as (%) change as compared to the control ± *SEM*. Statistical comparisons were made using one‐way ANOVA/Dunnett's multiple comparison test. Note (*) indicates a statistically significant difference when compared with controls

The mitochondrial location of MAO has also been reported (Lehninger, 1975), and MAO is used as a marker enzyme to indicate the presence of the outer membrane in the mitochondria. Ageing significantly decreased monoamine oxidase activity in the juvenile (*p* = .02) and adult (*p* = .01) as compared to the neonates (Figure 5, *n* = 5, *p* < .05).

## 4 DISCUSSION

Age‐dependent elevation in mitochondrial oxidative stress can contribute to decrease in testicular function. In this study, we report that ageing increased markers of oxidative stress/redox biomarkers such as ROS and nitrite, decreased mitochondrial complex activity, decreased antioxidant enzymes and promoted oxidation products formation in goat testis. Compelling evidence has shown that ageing can promote oxidative stress and lead to decreased function in various organs as well as several pathological conditions (Pole, Dimri, & Dimri, 2016). However, there are minimal studies that have related the changes in the markers of oxidative stress/redox biomarkers and mitochondrial functions with ageing scrotum. To the best of our knowledge this is the first report describing the impact of ageing on oxidative stress and mitochondrial function in goat testis.

Ageing (the physiological process of becoming older) represents the accrual of anatomical, physiological, biochemical, hormonal, neurochemical, physical, social and mental changes in an animal or humans over a defined period of time (López‐Otín, Blasco, Partridge, Serrano, & Kroemer, 2013). Ageing (senescence) surges the susceptibility to age‐associated diseases, whereas genetics determines vulnerability or resistance between species and individuals within species (Jeck, Siebold, & Sharpless, 2012). Sexual functions of goats have shown to decline during ageing (Smith, Brown, & Parkinson, 2006).

In this study ageing induced significant and profound increase in oxidative stress in addition to noteworthy decrease in the mitochondrial function in the goat testis. During the process of ageing, there was an increase in the production of reactive oxygen species and nitrites. The results obtained from this study indicate that the potential source of ROS formation is Complex I. Mitochondria acts as major site of ROS production, wherein electrons are transferred through the electron mitochondrial chain (ETC) in order to decrease the molecular oxygen. Similar to our results, other studies have reported that decreased activity of electron transport chain (ETC) complexes increase mitochondrial ROS production (Murphy, 2009). In our study, we found a statistically significant reduction in Complex I activity in juvenile and adult goat testis with no significant changes in Complex IV activity. Mitochondrial ROS formation is inversely related to Complex I activity which might indicate that the mitochondrial ROS generation is primarily due to compromised Complex I, and to a lesser extent in Complex IV (Zorov, Juhaszova, & Sollott, 2014). Mitochondrial electron transport is an important subcellular source of ROS. Complex I, also, is an integral membrane complex of the electron transport chain that catalyses electron transfer from NADH to ubiquinone. Complex I is an important site of superoxide anion generation in mitochondria (Hansford, Hogue, & Mildaziene, 1997). The produced superoxide is then scavenged by the mitochondrial enzyme superoxide dismutase to produce H2O2. Therefore, a deficient complex I activity can be considered a potential source of ROS in ageing tissues (Zalewska, Ziembicka, Żendzian‐Piotrowska, & Maciejczyk, 2019). Although ROS production may not be critical factor for ageing (López‐Otín et al., 2013), they are more likely to exacerbate age‐related diseases progression via oxidative damage and interaction with mitochondria (Dias, Junn, & Mouradian, 2013).

During ageing, mitochondria—the primary source of ROS—are often subjected to oxidative damage at a level that supersedes the protective capacity of the antioxidant response. Toxic effects of ROS on cellular components lead to accumulation of oxidative damage which causes cellular dysfunction with age (Di Meo, Reed, Venditti, & Victor, 2016). Due to their reactivity, high concentrations of ROS can cause oxidative stress by disrupting the balance of antioxidant and prooxidant levels (Zuo, Hemmelgarn, Chuang, & Best, 2015). We found a statistically significant decline in antioxidant (glutathione) levels. A decline in antioxidant system leads to increased susceptibility to oxidative stress especially in elderly as there is a decline in the efficiency of the endogenous antioxidant systems. Hence, organs with high rates of oxygen consumption and limited respiration levels such as brain and heart, are highly susceptible to this phenomenon. This partially explains the high prevalence of cardiovascular diseases and neurological disorders in the elderly (Corbi et al., 2008). Similarly, the decline in antioxidant system in the testis could account for the decline in sexual and reproductive capacities. Interestingly we found an increase in the glutathione peroxidase activity in juvenile and adult goat testis which we believe could be attributed to the compensatory response.

The altered prooxidant‐antioxidant redox status is more likely triggered by net effect of low antioxidants and increased reactive oxygen species (Chung et al., 2009; Lennicke, Rahn, Lichtenfels, Wessjohann, & Seliger, 2015). An imbalance between prooxidant‐antioxidant redox status and the dysregulation of the immune system as seen in ageing may lead to the exaggerated systemic inflammatory response with activation of inflammatory mediators. Hence, chronic inflammation as seen in ageing may serve as a pathophysiologic association which converts normal functional changes to the age‐related degenerative diseases (Viola & Soehnlein, 2015).

This increase in the reactive oxygen species in the juvenile and adult goats may be associated with the innate (non‐specific) immune system. The respiratory burst associated with the innate immunity is a process that involves enzymes and produces several types of reactive oxygen species. The reactive oxygen species essentially impact cellular processes by affecting the cell signalling, transcription factor and translation regulator under the controlled physiological conditions which in turn affects the cell growth. However, excessive production and prolonged exposure of reactive oxygen species can induce lipid peroxides and protein carbonyls (Hauck & Bernlohr, 2016). In our study, we found a statistically significant increase in lipid peroxidation and protein carbonyl content in juvenile and adult goat testis. Lipid peroxidation has shown to affect the sperm survival and the sperm fertility (Guthrie & Welch, 2012). Lipid peroxidation‐induced decreased sperm motility is because of the reactive oxygen species‐induced lesion in ATP utilization or in the contractile apparatus of the flagellum and can result in testicular hypoplasia and infertility.

The mitochondrial theory of ageing is still deliberated as an extension of the free radical hypothesis. Given the close relationship between oxidative stress, inflammation and ageing, the oxidation‐inflammatory theory of ageing has been proposed: ageing is a loss of homeostasis due to a chronic oxidative stress. The consequent activation of the immune system induces an inflammatory state that creates a vicious circle in which chronic oxidative stress and inflammation feed each other, and consequently, increases the age‐related morbidity and mortality (Fuente & Miquel, 2009).

The health and lifespan of the cell is related to caloric restriction, functions of DNA, fibroblasts, mitochondria and oxidative stress. Nevertheless, there is a significant gap in the current literature in the veterinary health about the role of oxidative stress and mitochondrial functions and its pertinent effect on the scrotum of the domestic and farm‐raised animals. The results obtained from this study regarding the changes in the oxidative stress and mitochondrial functions can help with the production, maintenance and sustenance of the goats. The limitations of the study are that we evaluated only the redox homeostasis parameters. We intent to perform other proteomics, endocrine and biochemical markers to evaluate several age‐related changes in goat testis. Furthermore, we will evaluate markers of inflammation, apoptosis and histological sections to study the senescence‐related changes. This study will help in designing innovative and novel healthy diets to provide during ageing. Our study can further help to augment goat preservation by providing different age‐appropriate diet to goat.

## 5 CONCLUSION

Health and lifespan of goats has been related to oxidative stress and mitochondrial functions. There is a constant increase in the prooxidants and decreased antioxidants & ATP contents during ageing in goat testis. The results from this study clearly established the altered redox status and mitochondrial dysfunction during ageing in goat testis. Herein, we provide a strong basis for future mechanistic studies and therapeutic strategies to improve the well‐being of goats.

## AUTHOR CONTRIBUTION

**Mohammed Majrashi:** Data curation; Formal analysis; Investigation; Methodology; Validation; Writing‐original draft. **Ayaka Fujihashi:** Investigation; Methodology; Validation; Visualization. **Mohammed Almaghrabi:** Formal analysis; Investigation; Methodology; Validation; Visualization. **Maali Fadan:** Investigation; Methodology; Resources; Validation. **Eddie Fahoury:** Investigation; Methodology; Resources; Writing‐review & editing. **Manoj Govindarajulu:** Software; Visualization; Writing‐original draft; Writing‐review & editing. **Sindhu Ramesh:** Resources; Software; Writing‐review & editing. **Haley Beamon:** Conceptualization; Formal analysis; Funding acquisition; Methodology; Resources; Supervision; Writing‐original draft. **Chastity N Bradford:** Conceptualization; Funding acquisition; Methodology; Resources; Validation; Visualization. **Olga Bolden‐Tiller:** Conceptualization; Data curation; Formal analysis; Investigation; Resources; Validation; Visualization. **Muralikrishnan Dhanasekaran:** Conceptualization; Data curation; Formal analysis; Funding acquisition; Investigation; Methodology; Project administration; Supervision; Validation; Visualization; Writing‐original draft; Writing‐review & editing.
